# Comprehensive Genomic Survey, Evolution, and Expression Analysis of GIF Gene Family during the Development and Metal Ion Stress Responses in Soybean

**DOI:** 10.3390/plants11040570

**Published:** 2022-02-21

**Authors:** Intikhab Alam, Xueting Wu, Liangfa Ge

**Affiliations:** 1 Department of Grassland Science, College of Forestry and Landscape Architecture, South China Agricultural University, Guangzhou 510642, China; intikhabalam2013@gmail.com (I.A.); wuxueting@stu.scau.edu.cn (X.W.); 2 College of Life Sciences, South China Agricultural University, Guangzhou 510642, China; 3 Guangdong Subcenter of the National Center for Soybean Improvement, College of Agriculture, South China Agricultural University, Guangzhou 510642, China

**Keywords:** GIF genes, *Glycine max*, gene duplication, expression patterns, Cd stress, Cu stress

## Abstract

The GIF gene family is one of the plant transcription factors specific to seed plants. The family members are expressed in all lateral organs produced by apical and floral meristems and contribute to the development of leaves, shoots, flowers, and seeds. This study identified eight GIF genes in the soybean genome and clustered them into three groups. Analyses of Ka/Ks ratios and divergence times indicated that they had undergone purifying selection during species evolution. RNA-sequence and relative expression patterns of these *GmGIF* genes tended to be conserved, while different expression patterns were also observed between the duplicated GIF members in soybean. Numerous cis-regulatory elements related to plant hormones, light, and stresses were found in the promoter regions of these *GmGIF* genes. Moreover, the expression patterns of *GmGIF* members were confirmed in soybean roots under cadmium (Cd) and copper (Cu) stress, indicating their potential functions in the heavy metal response in soybean. Our research provides valuable information for the functional characterization of each *GmGIF* gene in different legumes in the future.

## 1. Introduction

Soybean (*Glycine max* (L.) Merr) is one of the world’s most important crops, contributing to over 70% of the protein diet and 28% of vegetable oil consumption (http://soystats.com accessed on 25 December 2022). According to statistical evidence, global soybean production increased about 13-fold between 1961 and 2017. Compared to the dramatic increase in yield in rice, wheat, and maize over the last few decades (FAO data), the yield per unit area for soybean has not improved considerably, indicating the absence of a real green revolution in soybean. The economic value of soybean, as with other staple crop plants, is determined by the quantity (yield) and quality of seeds produced. Both are affected by various factors, including plant height, internode formation, number of branches, pods per plant, seeds per pod, and seed size [1,2]. Seed weight is a complicated characteristic, influenced by various hereditary and environmental influences. According to recent research, soybean seed weight heritability can reach up to 98%, implying that genetics is the most important factor in regulating phenotypic variability in soybean seed weight [3,4,5]. Understanding the genetic elements that regulate the traits of soybeans is critical to current efforts to increase soybean yield capacity and food quality. Several important genes, including the *GROWTH-REGULATING FACTOR* (*GRF*) genes, encode transcription factors that interact with the GRF-INTERACTING FACTOR (GIF) transcription cofactor to form a functional transcriptional complex [6,7]. In this unit, GIF operates to recruit SWI/SNF chromatin remodeling complexes to their target genes, where they can be activated or repressed by GRF [6,7]. In Arabidopsis, three GIF members, i.e., *ANGUSTIFOLIA3* (*AN3*; also known as *AtGIF1*), *AtGIF2*, and *AtGIF3*, lead to the formation of aerial organs in Arabidopsis [8,9,10]. GIFs are defined by the N-terminal domain homologous to the SNH domain of human SYNOVIAL TRANSLOCATION (SYT) [8,9,11]. *AtGIF1* single mutants develop smaller leaves and flowers due to a decreased cell count in Arabidopsis.

In contrast, an over-expression of *AtGIF1* develops a larger leaf owing to a rise in the cell count [8,9,12,13,14]. In rice, *OsGIF1* overexpression raises the leaves, stem, and seed size, whereas *OsGIF1* loss-of-function contributes to small plants [15,16]. In corn, *GIF1* mutants are dwarfs with thin leaves arising from a smaller number of cells [17]. *OsGIF1* interacts directly with *OsGRF4*, and its activation increases rice grain size [15]. GIF1 binds to the *unranched3* (*ub3*) gene promoter and regulates the expressions of multiple maize genes involved in shoot architecture and meristem development [8]. GIF directly interacts with GRF to regulate cell proliferation during leaf development [8,9]. The overexpression of *GIF* genes enhances organ expansion and can improve *GRF* activity [16,18,19]. In contrast, mutations in *GIF* genes mimic the halved organ size observed in *GRF* loss-of-function mutants or plants overexpressing *miR396* [8,9,20,21,22]. Moreover, simultaneous increases in Arabidopsis GRF3 and *GIF1* expression facilitate the production of larger leaf sizes than when these genes are expressed separately, implying that GRFs and GIFs combine to form a protein complex [23]. These studies have revealed that most GRF and GIF members are required to be studied redundantly for the cell proliferation of lateral organs, which determines the size of the final organs of different species.

This study provides a comprehensive analysis of the *GIF* genes in soybeans, including genome-wide identification, phylogenetic classification, chromosome location, syntenic relationships, gene structure, promoter cis-elements, and expression analysis in various tissues. Additionally, their responses to various heavy metal toxicity tests were analyzed. Our findings are important for understanding the function and evolution of GIF genes in legume species, as well as for future genetic engineering and crop improvement.

## 2. Results

### 2.1. Identification of GIF Proteins in Soybean

To identify each GIF protein family member in the soybean genome, we used the BLASTp search in phytozome plant databases. The identified redundant sequences were deleted, and the remaining nonredundant sequences were examined using the Pfam and SMART databases for the existence of a GIF domain. As a result of this screening, we retrieved eight GIF genes in the soybean genome, listed in Table 1. The theoretical isoelectric points, molecular weights, and amino acid encoding CDS sequence lengths of these identified GIF proteins were also given. These GIF proteins were generally small in size and ranged from 163 to 257 amino acids (aa) in soybean. The theoretical isoelectric points ranged from 4.84 to 6.03 for GIF proteins of soybean. The molecular weights ranged between 18.01 kDa and 27.48 kDa in soybean. The computed physiochemical properties calculated by the ProtParam tool predicted that almost all GIF proteins in soybean were hydrophilic (GRAVY < 0) and unstable (instability index > 40) but had thermal stability (aliphatic index = 50.01~71.13).

### 2.2. Phylogenetic Analysis

**Figure 1:**
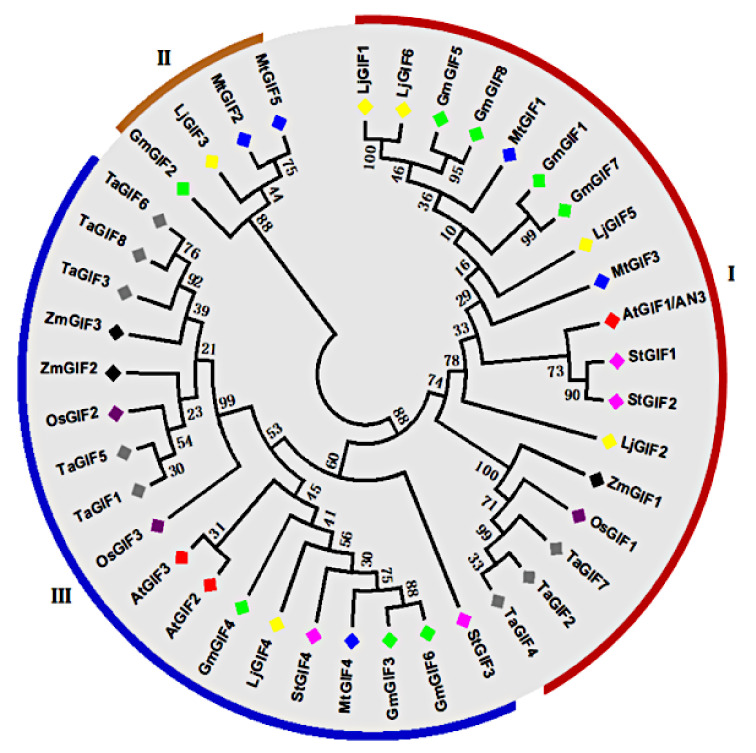
Phylogenetic tree of *A. thaliana*, *G. max*, *M. truncatula*, *O. sativa*, *T. aestivum, L. japonicas*, *S. toberosum*, and *Zea mays* GIF proteins. A neighbor-joining (NJ) phylogenetic tree was generated by MEGA7. The bootstrap values from 1000 replications are provided at each node.

**Figure 2:**
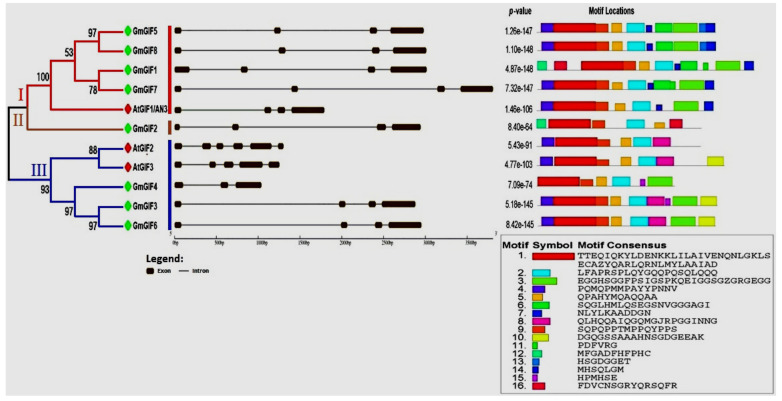
Schematic diagram of *A. thaliana* and soybean GIF gene organization and conserved motifs analysis. On the left side, the neighbor-joining (NJ) phylogenetic tree is shown, followed by the exons–introns, which are shown as black boxes and grey lines, respectively. On the right side of the picture, the neighbor-joining (NJ) phylogenetic tree is shown, followed by the various motifs depicted in various colors. Nonconserved sequences are indicated by black lines.

A phylogenetic tree was generated for the GIF genes from soybean and other species based on their encoding amino acid sequences using the MEGA v7 software by the neighbor-joining technique with 1000 bootstrap replicates. An extended phylogenetic tree based on the GIF protein sequences from different plant species, including *A. thaliana*, *Oryza sativa, Glycine max*, *Medicago truncatula*, *Lotus japonicas*, *Solanum toberosum, Triticum aestivum,* and *Zea mays,* was generated (Figure 1), showing the high degree of conservation between the homologous GIF members from different species. Moreover, GIF genes from the two species were clustered into three groups. GroupI contained one *A. thaliana AtGIF1/AN3* gene and four soybean genes, namely *GmGIF1*, *GmGIF5*, *GmGIF7*, and *GmGIF8*. GroupII contained one soybean gene (*GmGIF2*), and GroupIII contained two *A. thaliana* (*AtGIF1* and *AtGIF2*) and three soybean genes, including *GmGIF3*, *GmGIF4*, and *GmGIF6* (Figure 2).

### 2.3. Gene Structure and Motif Composition Analysis

In order to determine the structural variation of the members of the soybean GIF gene family, we examined the exon–intron structure of each soybean and Arabidopsis GIF gene according to the phylogenetic classification (Figure 2). We can observe that the exon numbers of GmGIF genes ranged from three to six, and the intron numbers ranged from two to five for two species. The gene structures for groupI were globally conserved within the two species, except for intron lengths, as soybean genes have more intron lengths than Arabidopsis. For groupIII members, there were various exons and introns within the two species, and differences in exon or intron length were also observed between orthologous members.

### 2.4. Structural Diversity of GIF Proteins

In order to characterize the structural diversity of GIF proteins from the two species, we analyzed their preserved motifs using the program MEME tool (Figure 2). Sixteen preserved motifs were detected in these GIF proteins from the two species. The length of these motifs ranged from 6 to 50 amino acids. Motif 1 and motif 2 were detected in all members’ GIF domains, while motif 3 was present in all except one soybean member (*GmGIF2*) in GIF proteins. Motifs 4–16 were shared each by a varied number of GIF gene members and can sometimes distinguish the members of different subfamilies or species. To better view the degree of sequence preservation between the members of the GIF gene family in soybean and Arabidopsis, motif sequences were generated for the GIF protein region containing GIF domains (Figure 2). We can observe that numerous residues in GIF domains were highly conserved within each of the two GIF gene members and between the two species.

### 2.5. Chromosomal Distribution and WGD Events in Soybean

**Figure 3:**
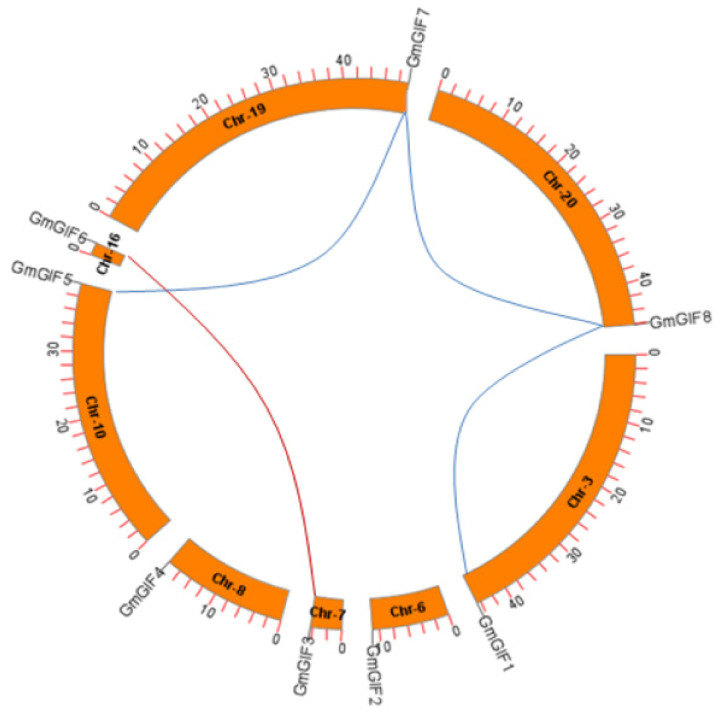
Distribution of GmGIF genes on eight chromosomes of soybean. The GmGIF genes are distributed throughout the chromosomes’ conserved collinear blocks. The number of chromosomes (Chr3, Chr6-Chr8, Chr10, Chr16, Chr19, and Chr20) is labeled in the center of each chromosome. The gene name and physical location (Mb) are shown on the upper positions of different chromosomes. The duplicated genes’ positions are connected to the same color lines as the corresponding chromosomes.

The chromosome location data of each soybean GIF gene were downloaded from the Phytozome database (Table 1), based on which these GIF genes were mapped on their corresponding chromosomes (Figure 3). As a result, the eight soybean genes were mapped on eight of the 20 soybean chromosomes (i.e., Chr 03, Chr 06-Chr 08, Chr 10, Chr 16, Chr 19, and Chr 20) (Figure 3). Gene duplication events are thought to be one of the major factors that contributed to the expansion of gene families during the genome’s expansion [24]. Soybean is an ancient tetraploid that has undergone two whole genome duplications [25]. The majority of soybean genes are paralogous genes, meaning they have multiple copies. According to the phylogenetic tree results and the plant genome duplication database (PGDD), the duplicated GIF gene pairs in soybean were determined. Three pairs of GmGIF genes were involved in segmental duplication (Figure 3). To explore the evolutionary selection type of these identified GmGIF genes, the Ka, Ks, and Ka/Ks ratios of all the three groups and between groups were computed (Table 2). The Ka/Ks ratios of groupI ranged between 0.1798 and 0.3707, with an average of 0.309. The estimated divergence times between the segmental duplication of groupI gene pairs ranged between 9.64 and 65.492 MY, with an average of 45.694 MY. In groupIII, the Ka/Ks ratios between gene pairs ranged from 0.0328 to 0.4521, with an average of 0.302, while the estimated divergence times of segmentally duplicated gene pairs in groupIII was 11.541 MY (Table 2).

### 2.6. Putative Cis-Regulatory Element Analysis

**Figure 4:**
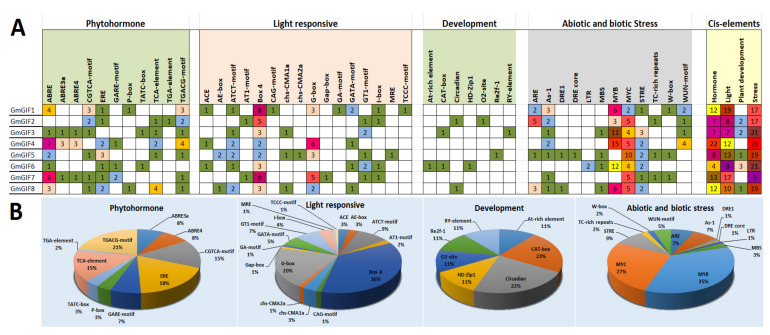
Analysis of a putative cis-acting element in the upstream region of GmGIF genes. (**A**) The numbers of distinct putative cis-acting factors discovered in the upstream region of GmGIFs in soybean. (**B**) Different grid colors showing GIF genes. A separate colored histogram is provided on the upper right side for the sum of the cis-acting elements in each group. Pie charts represent the ratio of each detected cis-acting factor in each group.

In order to study the types and distributions of cis-regulatory elements in the promoter, we retrieved the 2-kb upstream DNA sequences of soybean GIF genes from the phytozome database. The respective promoter regions were then subjected to the PlantCARE database for putative cis-elements analysis. Overall, 340 putative cis-acting elements from eight soybean genes in the promoter area were detected. Based on their involvement in different biological functions, these putative cis-acting elements were classified into four main groups, including phytohormone cis-elements (11), light-responsive cis-elements (16), plant growth and development cis-elements (7), and stress-responsive cis-elements (12) (Figure 4A). Three phytohormone-responsive cis-elements (i.e., ABRE, TGACG, and CGTCA involved in abscisic acid, auxin signaling, and methyl jasmonate), three light-related cis-acting elements (i.e., G-box, GT1, and Box 4), and three stress-responsive cis-elements (i.e., ARE, MYB, and MYC) were identified with a top ratio in the upstream regions of soybean *GIF* genes (Figure 4B). However, compared to the other three categories, the detection rate of cis-regulatory elements associated with growth and development was relatively low.

### 2.7. Expression Analysis of GIF Genes in Soybean

**Figure 5:**
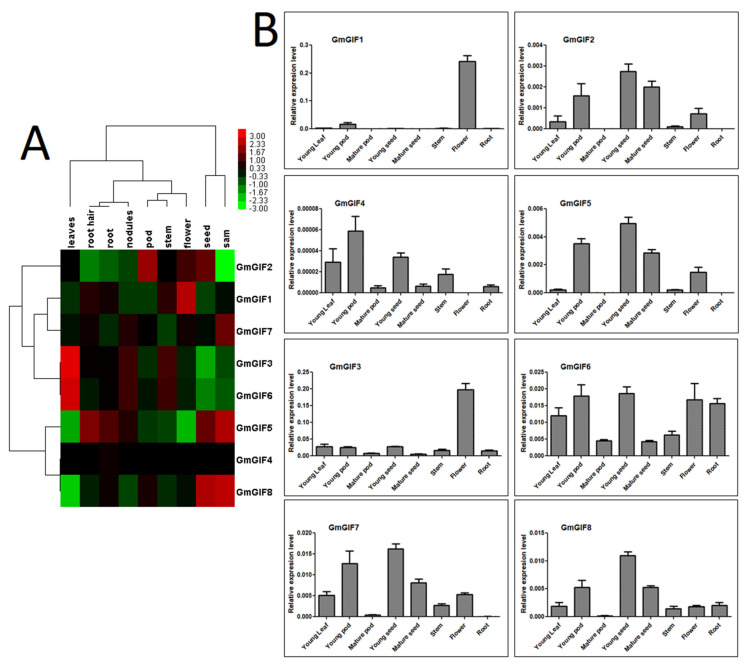
The soybean GIFs gene expression patterns in different tissues. (**A**) The Phytozome V12.1 database was used to obtain data on the expression pattern of eight GmGIF genes across various tissues. The color scale bar and the tissue types are listed on top. The gene names are shown on the right side of the heatmap. The relative signal value level is shown at the top of the heatmap. (**B**) RT−qPCR data on the expression patterns of eight soybean GIF genes across numerous tissues. The *x*-axis represents the time points, and the *y*-axis shows the relative expression level.

To obtain information about the expression patterns of the soybean GIF genes, we retrieved and analyzed their RNA-seq data from the Phytozome V12.1 database. The expression data (FPKM) were log2-transformed, clustered heat maps displaying the expression patterns of eight GIF genes across various tissues were generated (Figure 5A), and the relative expression of eight GIF genes across different tissues was further investigated using the real-time quantitative PCR (qRT-PCR) technique (Figure 5B). We can observe that all GmGIF genes were differentially expressed in all tissues or organs, except the *GmGIF4* gene, which was very lowly expressed. On the other hand, GmGIF genes specifically expressed in one or more tissues or organs were also observed in the soybean crop. For example, *GmGIF1* was substantially expressed in flowers, and *GmGIF7* was substantially expressed in SAM. Moreover, *GmGIF3* and *GmGIF6* were highly expressed in leaves, while *GmGIF5* and *GmGIF8* were highly expressed in the seed and SAM tissue (Figure 5A,B). Furthermore, these GIF genes were confirmed by qRT-PCR, and mostly similar expression patterns were observed between RNA-seq data and qPCR (Figure 5A,B).

### 2.8. Expression of GmGIFs in Response to Heavy Metal Stresses

**Figure 6:**
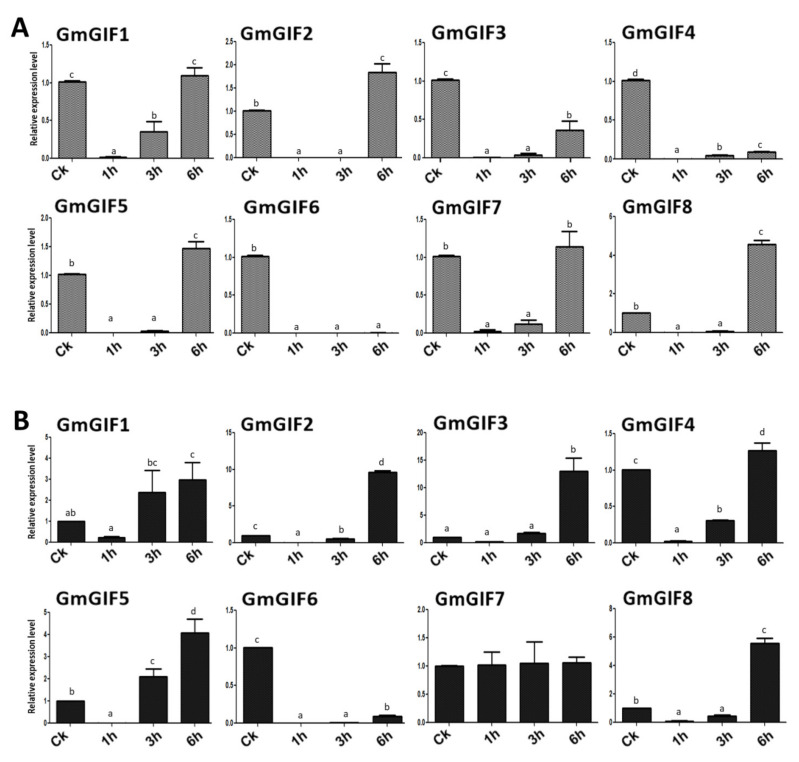
Relative expression patterns of *GmGIFs* genes under Cd and Cu treatment by RT−qPCR (**A**,**B**). The *x*-axis represents the time points, and the *y*-axis represents the relative expression scale. Tukey’s tests were used to examine differences between effects on different time hours under various treatments, and different letters show a significant difference (*p* < 0.05).

Numerous GRF genes bind with the transcription cofactor GIF, forming a functional transcriptional complex [6]. In this unit, GIF operates to recruit SWI/SNF chromatin remodeling complexes to their target genes so that they can be transcriptionally activated or inhibited by GRF. The expression of GRF is post-transcriptionally inhibited by microRNA (miR396) [6]. More specifically, miR396–GRF/GIF modulates many essential traits for plant growth that could influence agriculture production. Furthermore, in many species, the microRNA (miR396) and GRF gene expression have been revealed to be responsive to various stresses, including Cd stress [26,27,28,29,30,31]. Therefore, there is a need to examine the potential roles of GmGIFs in heavy metal stress responses. The expression of all these genes was quantified by qRT-PCR in soybean seedlings subjected to high levels of Cd or Cu ions. In general, GmGIF members’ expression was more vulnerable to Cu toxicity, followed by Cd toxicity. The expression of three GmGIF genes was enhanced by excess Cd, with two of them, namely *GmGIF2*, and *GmGIF8*, being 1–3 fold upregulated at 6 h of treatment, while three GmGIF genes, including *GmGIF3*, *GmGIF4*, and *GmGIF6*, were downregulated at all hours of Cd stress treatment, while mostly GIF genes were downregulated at 1–3 h of treatment and had enhanced expression with increasing hours of Cd treatment (Figure 6A). In Cu stress treatment, the expression levels of six genes were greatly enhanced, while four genes, namely *GmGIF2*, *GmGIF3*, *GmGIF5,* and *GmGIF8*, were significantly upregulated at six hours of treatment (Figure 6B).

## 3. Discussion

Plants have experienced extra genome duplication events as compared to other eukaryotes. Following duplication events, three functional consequences have occurred: gene loss, neofunctionalization, and subfunctionalization [32]. In duplication events, such as tandem and segmental duplications [33], the gene number increases, which may play a role in gene family evolution and genetic systems [33,34]. The GIF gene family is a group of plant transcription factors that play significant functions in the development of leaves, shoots, flowers, and fruits and are expressed in all lateral organs produced by apical and flower meristems [8,9,12,13,14,15]. The GIF gene family has been identified in various plant species, especially *A. thaliana* [9,12], *B. rapa* [35], rice [16], tomato [36], and *Zea mays* [37], and no study has been carried out in legume species, including soybean. In the current study, eight GIF genes were identified encoding the SSX2 domain in the soybean genome, which was much more than in other plants (Table 1). This number (8) is almost thrice that (3) of *A. thaliana*, which might be associated with whole-genome duplication [33]. Plant genomes have a large number of duplicate genes due to ancient duplication events and a high retention rate of existing pairs of duplicates [38]. Gene duplication is a main factor in gene family expansion and the evolution of distinct functions such as stress adaption and disease induction [24,38,39]. The main duplication patterns for gene family expansion are tandem and segmental duplications [40]. Segmental duplications more frequently occur as most plants’ genomes contain an abundance of duplicated chromosomal blocks due to polyploidy and chromosome rearrangements [41]. In the current study, six GmGIF genes underwent segmental duplication. Previously, studies have reported that soybean genomes have experienced two rounds of segmental duplication over their evolutionary history, about 13 and 59 Mya, resulting in about 75% of the genes being present in many copies [33]. The divergence time of GmGIF segmentally duplicated pairs diverged from 9.64 to 65.49 MY, with an average of 45.69 MY (Table 2). The Ka/Ks of all the segmentally duplicated gene pairs were found to be between 0.0328 and 0.3707, with an average of 0.2.79, suggesting the influence of purifying selection on the evolution of these gene pairs because a pair of genes having Ka/Ks < 1 could indicate purifying selection enforcing on the different protein-coding genes during evolution [42]. Our phylogenetic (Figure 1) analyses showed that, for each *A. thaliana* GIF gene, the corresponding orthologous genes were present with varied copy numbers. In our gene structure analyses, all the GmGIF genes’ members contained four exons and mostly similar exon/intron structural organization; the same results were also detected in tomato [36]. However, *AtGIF1/AN3* clustered in group1 had 4 exons and a similar exon structure in both species, except the *GmGIF1* exon had more length, whereas in group III, *AtGIF2* had six and *AtGIF3* had five exons [43], while in soybean, GIF genes contained 3–4 exons with different exon–intron lengths and structural organization (Figure 2). The motif composition of GIF domains showed that the mostly GIF genes were conserved between soybean and Arabidopsis, while some additional motifs were also identified in some GIF members (Figure 2). This shows that most GIF genes may conserve their basic cellular functions in Arabidopsis and soybean, while some others may have diverged from their primary functions or gained new functions during the evolution of the plant. Our promoter analysis revealed the presence of a significant number of phytohormone responsive elements (ABRE, CGTCA, and TGACG), and light-responsive (G-box, GT1, and Box 4) and stress-responsive (ARE, MYB, and MYC) cis-acting elements in the promoter region of these GIF genes in soybean (Figure 4), indicating that internal hormones and environmental signals can regulate the expression of these *GmGIF* genes. This result is consistent with previous research in tomato [36], where the GIF gene members were shown to be responsive to abiotic stresses. Furthermore, the majority of GIF gene promoters, including *GmGIF1*, *GmGIF4*, *GmGIF5*, *GmGIF7*, and *GmGIF8*, contain a typical G-box sequence (5′-CACGTG-3′), and it was previously reported that the KIX-PPD-MYC complex binds to the GIF1 promoter’s G-box sequence, suppressing expression and promoting seed development [44,45]. In many plant species, GIF genes play a significant role in the formation of leaves and seed development [16,44,46]. In *A. thaliana*, three GIF genes, i.e., *AtGIF1/AN3*, *AtGIF2*, and *AtGIF3*, have redundant functions and play important functions in developing vegetative and reproductive organs [12,13,14,23,47,48]. The GIF gene members were doubled (becoming eight) in soybean (Figure 1) and then constituted interesting candidates for genetic improvement of yield in the legume species. In the current study, expression profiles of genes at different stages of soybean development were analyzed (Figure 5A,B). We can observe that the expression patterns of these genes tended to be conserved between *G. max* and Arabidopsis, but different expression patterns were also observed between or among the duplicated members, indicating that their functions may have diverged, as in the study of maize [12,17,37]. For example, *GmGIF1* showed a clear preferential expression in flowers and a very low expression in seeds compared to their orthologs in rice; *OsGIF1* overexpression raised the size of the leaves, stems, and seeds. In contrast, *OsGIF1* loss-of-function contributed to small plant patterns [34,49], while *OsGIF1* interacts directly with *OsGRF4*, and its activation increases rice grain size [34]. The other two GmGIF members (*GmGIF5* and *GmGIF8*) showed a clear preferential expression in seed, suggesting a possible role in seed development, as reported for the other species’ GIF genes function in cell proliferation to determine seed size [15,16,44].

Heavy metal contamination has emerged as one of humanity’s most critical problems [50]. When concentrations of heavy metals in the soil are above a specific level, plant photosynthesis is limited, and absorption of nutrients is insufficient, severely limiting plant production and quality [49,51]. The highly concentrated heavy metals in plants can pass through the food chain, affecting the health of animals and humans [52]. Cadmium (Cd) is one of the most poisonous heavy metal pollutants to animals and plants due to its high mobility and toxicity among all heavy metal contaminants. Cd toxicity alters plant cellular functions; it reduces root growth, disrupts regulatory systems, causes oxidative stress, impedes nutrient acquisition, destroys membranes, and may induce cell death in severe toxicity situations [53,54]. To examine the potential role of the GmGIF genes in Cd stress, all the GmGIF genes were used for qRT-PCR in soybean seedlings subjected to high levels of Cd stress. The expression level of most GmGIF genes remained constant or changed slightly, except for *GmGIF2* and *GmGIF8*, which exhibited 1–3 fold upregulations at 6 h of treatment (Figure 6A). As previously reported, there is a crucial role of miR396 in various species, including canola, maize, radish, and soybean crops under Cd stress [26,27,28,29,30,31], as well as the expression profile of GRF target genes in response to Cd stress [31], so it will be very interesting to conduct further functional studies on the miR396–GRF/GIF module, which plays a crucial role in a plant’s response to various stresses.

Cu ion concentration is a critical element impacting a variety of metabolic pathways involved in plant growth and development. Cu deficiency or excess can impact critical metabolic processes in vivo, including impairing plant development [55]. Although plants obtain Cu primarily from the soil, roots play a significant role in Cu bioavailability. However, this mechanism varies by species and soil [56]. Cu deficiency alters the architecture of plant leaves and roots, drastically reducing chlorophyll and photosynthesis [57]. Cu ions inhibit the uptake and storage of other nutrient elements, a phenomenon known as “metal poisoning” [58]. As Cu ions poison plants, the highest concentration of Cu is found in the roots, followed by the buds and leaves [55]. In this study, all GmGIF genes were used for qRT-PCR in soybean seedlings subjected to high levels of Cu stress. The results revealed that Cu stress induced the expression of mostly GmGIFs genes in the root. Remarkably, the expression levels of *GmGIF2*, *GmGIF3*, and *GmGIF8* in plant roots increased more than fivefold after 6 h of stress (Figure 6B). The root organ is more sensitive to Cu stress than the other organs, which might be initiated by Cu ion uptake in plants. The root organ first takes the Cu ion and transports it into the xylem and phloem via various transporters [55]. In general, *GmGIF* genes’ expression was relatively higher; thus, the significant reduction in the roots is good evidence of Cu toxicity. As a result, the findings of this work are valuable for further validation of *GmGIFs* function in response to Cu stress, which is likely to be exploited to improve soybean plant resistance to Cu stress.

## 4. Materials and Methods

### 4.1. Identification of GIF Proteins in Soybean

Many approaches were used to determine the complete set of GIF protein genes in soybeans. First, all Arabidopsis GIF protein sequences were obtained from the TAIR database (http://www.arabidopsis.org accessed on 2 February 2022) and utilized as a query in BLASTp searches against the phytozome soybean database. Secondly, GIF domain sequences of Arabidopsis, rice [16], and maize [32] were used as a query for BLASTp searches against the same soybean databases. The obtained sequences were then analyzed using the Interpro database (http://www.ebi.ac.uk/interpro/ accessed on 5 February 2022), SMART tool database (http://smart.embl-heidelberg.de 5 February 2022), and Pfam database (http://pfam.sanger.ac.uk accessed on 5 February 2022) to ensure the presence of GIF-related motifs in the related arrays. The ProtParam tool (http://web.expasy.org/protparam/ accessed on 5 February 2022) was used to determine the molecular weight (Wt) and theoretical isoelectric point (pI) of the GmGIF proteins.

### 4.2. Phylogenetic Analysis

The sequences of GIF proteins were aligned using ClustalW and a phylogenetic tree was designed using the MEGA v7 software using the neighbor-joining (NJ) technique with 1000 bootstrap replicates [59].

### 4.3. Gene Structure and Motif Analysis

The exon–intron regions of GmGIF proteins were analyzed using the Gene Structure Display Server v2.0 (http://gsds.cbi.pku.edu.cn/ accessed on 8 February 2022). The MEME tool (version 4.11.4; http://meme-suite.org/tools/meme accessed on 10 December 2022) was used to classify and analyze the conserved motifs of GIF proteins. The online WebLogo tool was used to create sequence logos based on the conserved motif alignments.

### 4.4. Analysis of GmGIF Promoters in Soybean

The promoter regions of all GmGIF genes (2-kb upstream sequences from the start codon (ATG)) were retrieved from the Phytozome database (https://phytozome.jgi.doe.gov accessed on 10 December 2022). Afterward, the putative generic files were submitted to the Plant-CARE database (http://bioinformatics.psb.ugent.be/webtools/plantcare/html/ accessed on 10 December 2022) for determination of cis-acting elements.

### 4.5. Chromosomal Location and Duplication of GmGIF Genes in Soybean

The Phytozome database was used to obtain the chromosome location data for each soybean GIF gene. Using the TBtools program, the genes were mapped to their respective soybean chromosomes [60]. Differently colored lines were used to indicate genes that were segmentally duplicated.

### 4.6. Gene Duplication and Ka/Ks Values Calculation

According to the phylogenetic tree results and searching of the plant genome duplication database (PGDD) [61], we determined the duplication of potential soybean GIF genes on the segmentally duplicated regions. We estimated the nonsynonymous ratios (Ka), synonymous ratios (Ks), and evolutionary constraints (Ka/Ks) between the soybean GmGIF genes in order to identify pairwise combinations of genes encoding proteins with altered functions [62] by using PAL2NAL and codeml in the PAML package (http://www.bork.embl.de/pal2nal/index.cgi?example=Yes#RunP2N accessed on 15 January 2022) [63]. The divergence time was determined by using the formula T = Ks/2R, Ks/ (2 × 6.1 × 10^−9^) × 10^−6^ MYA (million years ago), where T represents the divergence time, Ks represents the synonymous substitutions/site, R represents the rate of nuclear gene divergence in plants, and the R-value is defined as 6.1 × 10^−9^ synonymous substitutions/site/year in the case of dicotyledonous plants [42].

### 4.7. Expression Patterns of GIF Genes in Soybean

The RNA-seq data for gene expression in various soybean tissues (root, root hair, nodules, stem, sam, leaf, flower, pod, silique, and seed) were obtained from the Phytozome V12.1 database (https://www.phytozome.jgi.doe.gov/pz/portal.html accessed on 15 January 2022). Cluster expression software v3.0 (http://bonsai.hgc.jp/mdehoo/n/software/cluster/ accessed on 15 January 2022) was used to assess the expression level (Fragments/Kilobase of Exon Model/Million mapped, FPKM) of identified soybean GIF genes. FPKM values were log2-transformed, Euclidean distances were calculated, and the clustering average linkage was used. The clustering tree and gene expression heat map were created using the Java Tree View software (version 1.1.5r2, http://jtreeview.sourceforge.net/ accessed on 15 January 2022).

### 4.8. Plant Materials and qPCR Analysis

Soybean cv. Williams 82 seeds were surface-sterilized with 1% sodium hypochlorite for 5 min and moderate shaking and then washed with ddH_2_O. The sterilized seeds were then planted in sterilized soil and sand mixture-filled containers (soil: sand = 1:1) and grown in an artificial growth compartment with a photoperiod of 16 h light and 8 h dark at 22 °C and humidity at 65–70%. To investigate possible functions of GmGIFs in response to heavy metal stresses, 7-day-old seedlings were treated for 1–6 h with excess Cd (50 µM of CdCl_2_) and Cu (50 µM of CuSO_4_·5H_2_O) treatments. Three biological replicates were used for each treatment. Following the manufacturer’s instructions, total RNA was extracted from each soybean sample’s frozen roots (0.3 cm) using a plant RNA extraction kit (OMEGA, Guangzhou, China). The RNA quality was determined by gel electrophoresis and the NanoDrop 2000 Spectrophotometer (Thermo Fisher Scientific, Waltham, MA, USA). The cDNA Synthesis Kit (Takara, Dalian, China) was used to make first-strand cDNA from 1 μg of total RNA of each sample. Prior to analysis, the reverse transcription products were diluted 20-fold and kept at 20 °C. Primer3Plus software (http://www.primer3plus.com/ accessed on 10 November 2022) was used to generate gene-specific primers for GIF genes in soybean. Specific primers for GIF genes were used in qRT-PCR, and Actin primers for soybean were used as a control (Table S1). The qRT-PCR was carried out on a Bio-rad CFX Connect™ Real-Time System. The experiment was carried out in a total volume of 20 μL, which included 2 μL of the cDNA template, 0.8 μL of forward and reverse primers (10 μM), 10 μL of the ChamQTM Universal SYBR qPCR Master Mix (Vazyme, Q711–02), and 6.4 μL of sterile distilled water. Three replicates were operated for each sample to calculate the average Ct values. The collected values were analyzed using the 2^−ΔΔCt^ methods. The relative expression levels of each sample were normalized by housekeeping genes (*GmActin-2*).

## 5. Conclusions

In conclusion, eight GIF genes were respectively identified in soybean genomes. These genes were clustered into three groups in each species by phylogenetic analysis. Analyses of gene structure, motif composition, and sequence logos showed that the GIF genes were highly conserved between soybean and Arabidopsis. Analyses of Ka/Ks ratios and divergence times indicated that most of the GIF gene members had undergone strong purifying selection during species evolution, and their basic functions may be well conserved. RNA-seq and relative expression patterns of these GIF genes tended to be conserved. However, different expression patterns were also observed between or among the duplicated members in soybeans, indicating that their functions may have also diverged. Promoter analysis revealed the existence of a significant number of phytohormone-, light-, and stress-responsive cis-acting elements in the upstream regions of these GIF genes in soybean. The expression profile of GmGIF genes in response to Cd or Cu stress revealed that members of this family are greatly involved in metal ion transport, particularly root transport of Cu ions. As a result, our research contributes to the functional determination of each GIF gene across legume species and may support the genetic engineering of some of these GIF gene members that are important for plant growth, development, and stress response in legume species.

## Figures and Tables

**Table 1:** Gene list and physicochemical and biochemical characteristics of GIF genes in soybean.

Gene_ Name	Gene_Locus	e_Value	Chromosome	Start	End	Strand	Length (CDS)	Protein Length (aa)	Molecular Weight (kDa)	Isoelectric Point (pI)	Atomic Composition	Instability Index (II)	Aliphatic Index	Gravy
*GmGIF1*	Glyma.03G249000	4.45 × 10^−57^	Chr03	44521820	44526089	Positive	774	257	27.48	6.03	3751	76.2	61.56	−0.507
*GmGIF2*	Glyma.06G134400	8.63 × 10^−21^	Chr06	11045274	11048495	Reverse	588	195	21.5	4.84	2975	55.95	71.13	−0.585
*GmGIF3*	Glyma.07G051300	4.67 × 10^−65^	Chr07	4426134	4430143	Positive	642	213	22.57	5.53	3085	67.22	52.07	−0.716
*GmGIF4*	Glyma.08G221100	4.5 × 10^−28^	Chr08	17965240	17966422	Positive	492	163	18.01	5.36	2470	55.33	65.28	−0.591
*GmGIF5*	Glyma.10G164100	1.85 × 10^−57^	Chr10	39822153	39825768	Positive	639	212	22.66	5.8	3099	70.46	64.95	−0.592
*GmGIF6*	Glyma.16G020400	6.16 × 10^−66^	Chr16	1854042	1858037	Positive	633	210	22.36	5.73	3053	68.76	50.01	−0.775
*GmGIF7*	Glyma.19G246600	1.92 × 10^−59^	Chr19	49323457	49327857	Positive	774	257	22.64	5.45	3091	61.4	60.95	−0.637
*GmGIF8*	Glyma.20G226500	9.2 × 10^−55^	Chr20	46058411	46062129	Positive	639	212	22.82	6.03	3118	70.66	61.75	−0.65

**Table 2:** The Ka, Ks, and Ka/Ks ratio within group or between groups and divergence time of segmentally duplicated GIF gene pairs in soybean.

Groups	Gene Pairs		Segmentally Duplicated	Ka	Ks	Ka/Ks	Duplication Date (MY)
	Gene Name	Gene Name					
GroupI	*GmGIF1*	*GmGIF5*	Yes	0.2741	0.799	0.343	65.492
	*GmGIF1*	*GmGIF7*	Yes	0.1037	0.2799	0.3707	22.943
	*GmGIF1*	*GmGIF8*	Yes	0.2584	0.874	0.2957	71.639
	*GmGIF5*	*GmGIF7*	Yes	0.1476	0.6515	0.2265	53.402
	*GmGIF5*	*GmGIF8*	Yes	0.0211	0.1176	0.1798	9.639
	*GmGIF7*	*GmGIF8*	Yes	0.1591	0.6228	0.2555	51.049
GroupI vs. groupII	*GmGIF1*	*GmGIF2*	No	0.8539	6.4413	0.1326	_
	*GmGIF5*	*GmGIF2*	No	0.8626	2.7244	0.3166	_
	*GmGIF7*	*GmGIF2*	No	0.902	44.9847	0.0201	_
	*GmGIF8*	*GmGIF2*	No	0.8429	48.8651	0.0172	_
GroupIII	*GmGIF3*	*GmGIF4*	No	0.2875	0.6843	0.4201	_
	*GmGIF3*	*GmGIF6*	Yes	0.0046	0.1408	0.0328	11.541
	*GmGIF4*	*GmGIF6*	No	0.2786	0.6162	0.4521	_
GroupI vs. groupIII	*GmGIF1*	*GmGIF3*	No	0.6706	7.8299	0.0857	_
	*GmGIF1*	*GmGIF4*	No	0.7318	10.5829	0.0692	_
	*GmGIF1*	*GmGIF6*	No	0.6634	48.1752	0.0138	_
	*GmGIF5*	*GmGIF3*	No	0.557	3.5068	0.1588	_
	*GmGIF5*	*GmGIF4*	No	0.6838	52.7005	0.013	_
	*GmGIF5*	*GmGIF6*	No	0.5457	2.7793	0.1964	_
	*GmGIF7*	*GmGIF3*	No	0.5732	4.8078	0.1192	_
	*GmGIF7*	*GmGIF4*	No	0.6586	54.035	0.0122	_
	*GmGIF7*	*GmGIF6*	No	0.5962	4.5136	0.1321	_
	*GmGIF8*	*GmGIF3*	No	0.5582	4.1385	0.1349	_
	*GmGIF8*	*GmGIF4*	No	0.6471	7.5525	0.0857	_
	*GmGIF8*	*GmGIF6*	No	0.5383	4.0524	0.1328	_
GroupII vs. groupIII	*GmGIF2*	*GmGIF3*	No	0.5734	2.2879	0.2506	_
	*GmGIF2*	*GmGIF4*	No	0.7968	5.2468	0.1519	_
	*GmGIF2*	*GmGIF6*	No	0.5519	1.8676	0.2955	_

## Data Availability

The datasets that support the conclusions of this article are included in this article.

## Supplementary Materials

The following are available online at https://www.mdpi.com/article/10.3390/plants11040570/s1, Table S1: Primer sequences for qPCR used in this study.
