# Supplementary material for: Comprehensive Genomic Survey, Evolution, and Expression Analysis of GIF Gene Family during the Development and Metal Ion Stress Responses in Soybean
Source: Plants (Basel). 2022 Feb 21;11(4):570. doi: 10.3390/plants11040570 (PMC8876841; doi:10.3390/plants11040570)
Supplement: Supplementary file 1 [file plants-11-00570-s001.zip › plants-1543887-supplementary.pdf]

Table S1. Sequences of GmGIFs gene primers for qPCR used in this study

Sr.no.	Gene name	Primer (5' - 3')
1	GmGIF1-F	ATCAGGCAGTGGCTCTGTAG
	GmGIF1-R	TTCACCACCCTCTGAGCTTC
2	GmGIF2-F	ATTTCCCAGTGTCCCTACCC
	GmGIF2-R	GCAAGTTTGGCCAGAAAAGT
3	GmGIF3-F	GCTGCTCTTGGAGGTGGA
	GmGIF3-R	CGTCTCCACCAGCTGTTCC
4	GmGIF4-F	GGCCTGTTATGACACATTGC
	GmGIF4-R	TGTTTGTGAATGCTGCAAATC
5	GmGIF5-F	GCTCCTGTACTCCCAACA
	GmGIF5-R	CCAATGCGTACAAAGTCC
6	GmGIF6-F	ACAATGAGGGCGGCAACA
	GmGIF6-R	AGCTGCAGAGCTGCCTTG
7	GmGIF7-F	GCAGCACGCTCATCTCTT
	GmGIF7-R	CCTCCTCCCACATTCACA
8	GmGIF8-F	AACTTGGCATGAGCTCCAAT
	GmGIF8-R	CTCGGCCTTCACCAGAAC
9	GmACTIN2-F	ACTGGAATGGTGAAGGCAGG
	GmACTIN2-R	CATTGTAAAATGTGTGATGCCAG
